# Implantable antenna for biomedical applications: electromagnetic, SAR, and thermal performance evaluation

**DOI:** 10.1038/s41598-025-21600-7

**Published:** 2025-10-10

**Authors:** Amr Shabana, Ahmed El-Bakry, Shimaa Mahdy

**Affiliations:** Department of Electrical Engineering, Egyptian Academy for Engineering and Advanced Technology (EAE&AT), Affiliated to the Ministry of Military Production, El-Nahda, Al Salam First, Egypt

**Keywords:** COMSOL, CST, Implantable antenna, Thermal analysis, Biological techniques, Biotechnology, Engineering, Health care, Medical research, Physics

## Abstract

This paper presents the design and evaluation of a miniature implantable antenna for biomedical applications, optimized for narrowband telemetry at 2.402 GHz within the ISM band. The antenna achieves ultra-compact dimensions of 4 × 3.5 × 0.254 mm3 and exhibits stable impedance matching with a reflection coefficient (S11) below − 22 dB and a bandwidth of approximately 10.4%. The realized gain of − 28.3 dBi lies within the expected range for implantable devices, ensuring reliable short-range communication. Safety and biocompatibility were comprehensively assessed using CST Microwave Studio and COMSOL Multiphysics, incorporating RF exposure analysis and the Pennes bioheat equation. Results confirmed compliance with SAR limits (0.385 W/kg for 1 g tissue at 1 mW input) and showed that thermal rise remained ≤ 2 K under continuous operation. Furthermore, ex vivo validation in bovine fat confirmed the antenna’s performance in a biological environment. Overall, the study provides a comprehensive evaluation of the antenna’s electromagnetic characteristics, SAR compliance, and thermal safety, demonstrating its suitability for implantable biomedical devices such as pacemakers, neurostimulators, and biosensors.

## Introduction

The rapid development of implanted medical devices has made wireless communication a critical component of modern healthcare. Implantable antennas enable bidirectional data transfer for telemetry, control, and diagnostics, making them key elements in Internet of Medical Things (IoMT) platforms and biomedical telemetry systems^1^.

Unlike conventional antennas, implantable antennas operate inside the human body, where surrounding biological tissues are lossy and non-uniform. These conditions cause signal attenuation, impedance mismatching, and frequency detuning. Therefore, implantable antennas must meet stringent requirements, including compliance with specific absorption rate (SAR) safety limits, biocompatibility, miniaturization, and stable performance under varying physiological conditions such as tissue heterogeneity, motion, and temperature changes^2,3^.

Additionally, implantable antennas are constrained by the small size and limited power of medical implants, making efficiency and bandwidth optimization crucial. Medically designated frequency bands such as MICS (402–405 MHz), ISM (2.4–2.48 GHz), and UWB (3.1–10.6 GHz) offer trade-offs between tissue penetration, antenna size, and data throughput^3,4^. The ISM band, in particular, is attractive for subcutaneous applications due to its license-free spectrum and compatibility with low-power telemetry standards.

Recent research has introduced various strategies to improve implantable antenna performance, including miniaturization techniques (e.g., meandered lines, high-permittivity substrates, metamaterials), computational modeling, and biocompatible materials. These advancements support a wide range of applications, such as in-body diagnostics, drug delivery, cardiac monitoring, and neural recording^3^. However, most existing works optimize only one aspect—either bandwidth, SAR, or compactness—while practical biomedical devices require a balanced design that addresses multiple performance parameters simultaneously.

Several designs have demonstrated compact multi-band operation. For example, compact antennas operating at MICS, ISM, and WMTS bands have been reported with dimensions as small as 3.5 × 3.5 × 0.26 mm^3^ and gains ranging from − 18 to − 42 dBi^5,6^. While these works highlight promising approaches, many exhibit limitations such as extremely narrow bandwidth, high SAR values exceeding safety limits, or a lack of validation across realistic tissue environments.

To address these challenges, the present study introduces an implantable antenna design that simultaneously considers radiation efficiency, gain, directivity, SAR, and thermal performance. Unlike prior works that mainly relied on a single simulator, this work combines CST Microwave Studio with COMSOL Multiphysics to provide a more accurate and holistic evaluation. The dual-simulation approach enables not only the electromagnetic analysis of the antenna but also detailed SAR and thermal assessments within biological tissues, offering a more realistic picture of device safety and performance.

This integrated methodology, rarely reported in the literature, ensures reliable wireless communication while complying with biomedical safety standards. The contributions of this work can therefore be summarized as follows:

1. Design of a compact implantable antenna optimized for the ISM band with sufficient margin to tolerate tissue variability and detuning.
2. Comprehensive evaluation of radiation characteristics, efficiency, and SAR within lossy tissue environments.
3. Dual-simulation methodology (CST + COMSOL) providing enhanced accuracy for both electromagnetic and thermal performance analysis.

By addressing efficiency, bandwidth, and safety in a unified framework, the proposed design advances the state of implantable antennas and enhances their applicability for subcutaneous biomedical devices.

## Methods

### The proposed antenna design

**Fig. 1:**
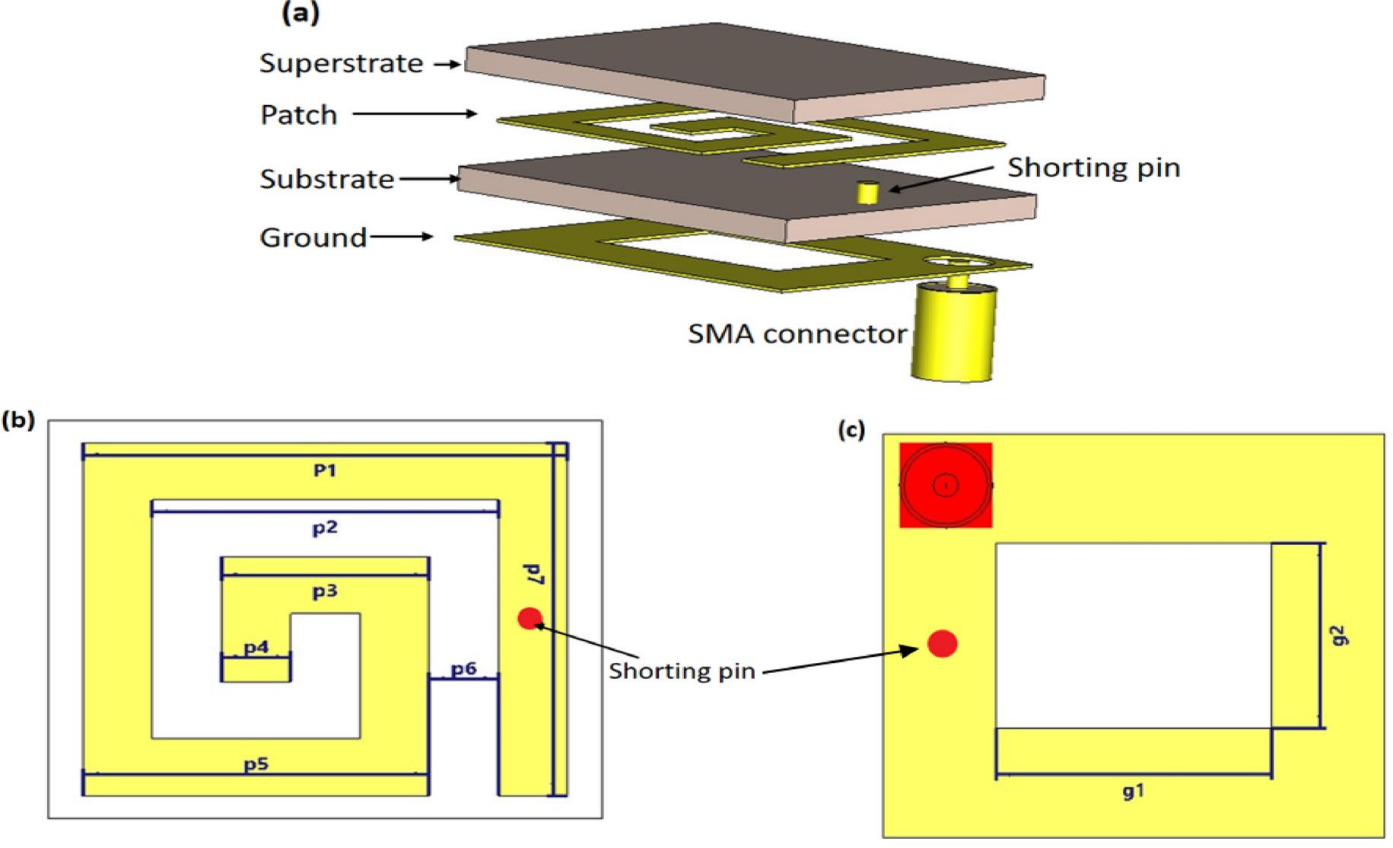
The proposed implantable antenna structure designed and simulated in CST (**a**) The proposed antenna layers, (**b**) the patch dimension, and (**c**) the ground dimension.

A rectangular patch structure is employed with various miniaturization techniques to achieve a compact antenna design with enhanced performance. Figure 1 illustrates the structure of the proposed implantable antenna, which has total dimensions of 4 × 3.5 × 0.254 mm3. Table 1 provides the specific dimensional parameters of the proposed antenna. Rogers RT/Duroid 6010LM, a material characterized by a high relative permittivity (ε_r_ = 10.2) and low loss tangent (tanδ = 0.0023) with a thickness of 0.25 mm, is used as both the substrate and superstrate. This material selection supports antenna miniaturization and provides electrical insulation from surrounding biological tissues for ex vivo experiments, but it does not fully substitute for in vivo biocompatibility testing. For in-body use, this substrate would be encapsulated with a thin biocompatible coating (e.g., silicone, parylene, or Teflon) to provide insulation and prevent direct interaction with body fluids or tissues^3^. In the proposed antenna design, a coaxial feed is employed and modeled as a lumped port to provide excitation, as shown in Fig. 1.

**Table 1:** The dimensions of the proposed antenna.

Parameter	p1	p2	p3	p4	p5	p6	p7	g1	g2
Dimension (mm)	3.5	2.5	1.5	0.5	2.5	0.5	3.1	2.2	1.6

The inclusion of a shorting pin in the compact antenna design plays a vital role in enhancing its performance for biomedical applications. Creating a direct electrical link between the radiating element and the ground plane reduces the resonance frequency without increasing the antenna’s dimensions. For implanted medical devices, this trait is beneficial in attaining compactness. The shorting pin also helps with impedance matching, which reduces signal reflections and improves power transfer efficiency. Also, the shorting pin improved the bandwidth and radiation properties by changing the current distribution inside the antenna. This makes it appropriate for applications such as the MICS band^7,8^.

**Fig. 2:**
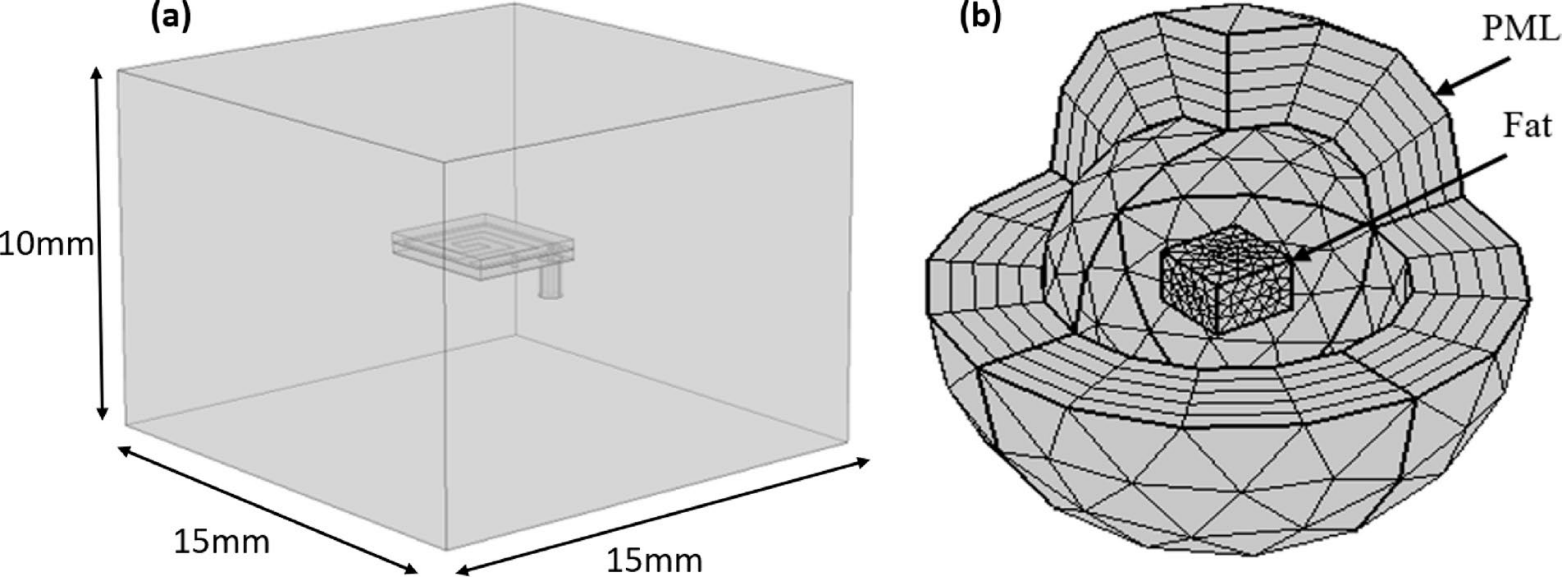
Simulation setup in COMSOL Multiphysics (**a**) The proposed antenna embedded within the human fat tissue model; (**b**) the mesh structure of the proposed simulation model.

The complete antenna structure is embedded at the center of a cuboid-shaped human fat tissue model with dimensions of 15 × 15 × 10 mm3, as illustrated in Fig. 2. This lossy medium is simulated using CST Microwave Studio and COMSOL Multiphysics. After constructing the proposed model, a mesh consisting of 50,510 domain elements, 8655 boundary elements, and 1417 edge elements is generated to apply the radiofrequency and bioheat physics across the respective domain, boundary, and edge regions. In our simulations, the Perfectly Matched Layer (PML) is implemented as a virtual domain that surrounds the physical region of interest to absorb outgoing wave energy in frequency-domain analyses. It is particularly effective in eliminating unwanted boundary reflections that are not caused by impedance mismatches. Acting like a skin-like absorber, the PML prevents wave reflections, and for optimal performance. The PML thickness should exceed the wavelength of the propagating wave to ensure effective absorption^9,10^.

CST Microwave Studio and COMSOL Multiphysics were employed for their respective strengths in electromagnetic and thermal simulations. CST was utilized to accurately model antenna parameters such as the reflection coefficient (S11), gain, and SAR, using solvers optimized for high-frequency electromagnetic analysis. COMSOL, with its multiphysics capabilities, enabled the integration of RF physics with bioheat transfer modeling, allowing realistic thermal simulations based on Pennes’ bioheat equation. This dual-platform strategy ensured a comprehensive evaluation of both the electromagnetic and thermal performance of the proposed antenna. To verify the simulation accuracy, results obtained from CST and COMSOL were cross-compared, and experimental measurements of the reflection coefficient (S11) were performed on ex vivo bovine fat samples.

**Fig. 3:**
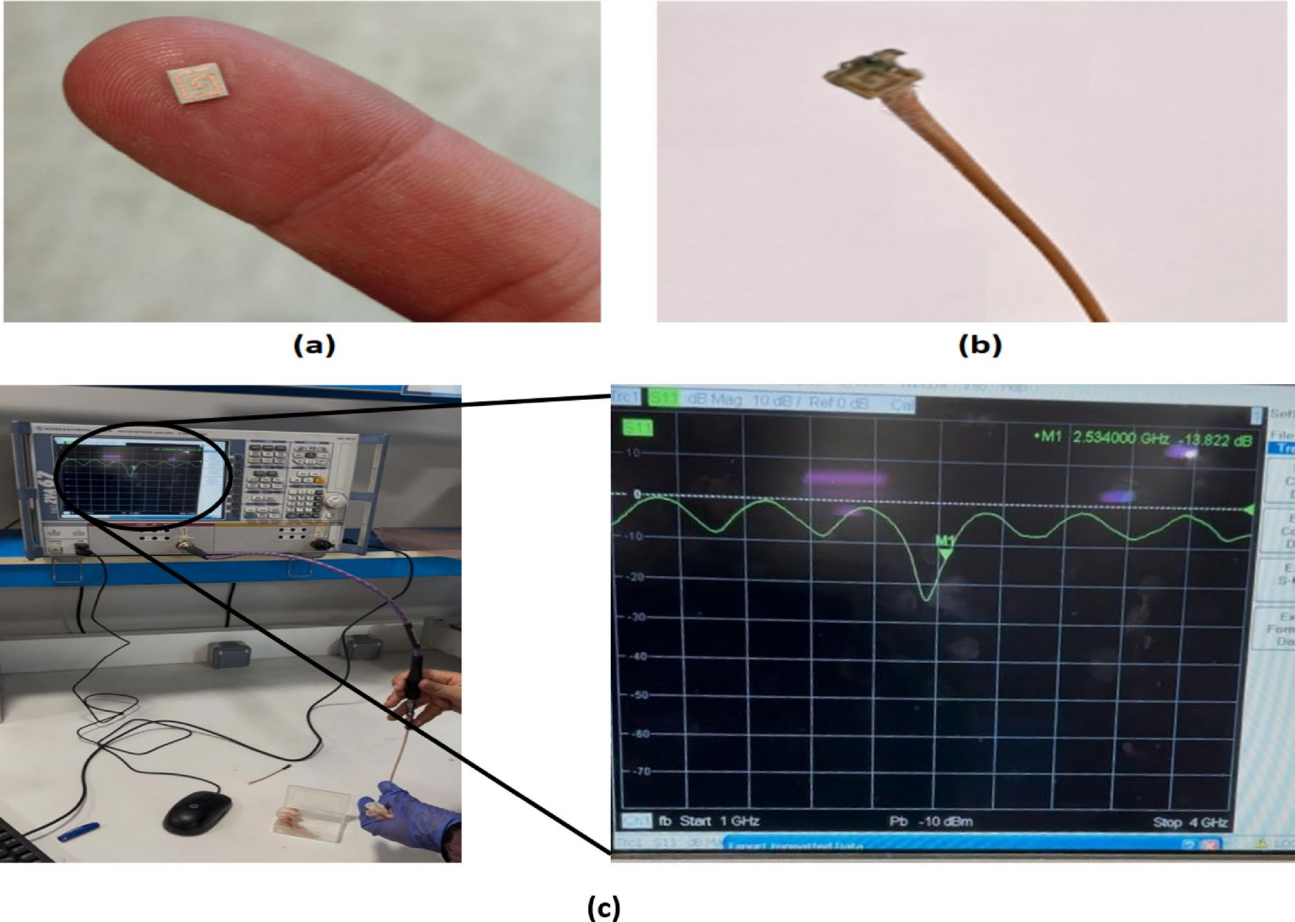
The fabricated proposed antenna: (**a**) without the SMA connector, (**b**) with the SMA connector and cable attached, and (**c**) positioned between two layers of bovine fat during the reflection coefficient (S11) measurement experiment.

Furthermore, the proposed antenna prototype was fabricated and experimentally tested at the Electronics Research Institute (ERI), Cairo. A vector network analyzer (VNA) was employed to measure the reflection coefficient (S11) of the fabricated antenna, as illustrated in Fig. 3. The measured results were then compared with the simulation outcomes to validate the design.

### Radiofrequency interaction with biological tissues

Radiofrequency (RF) propagation controls how electromagnetic waves interact with biological tissues, which are lossy and heterogeneous media. Because tissues’ dielectric characteristics change with frequency, these interactions influence the antenna’s performance in terms of efficiency, radiation pattern, and impedance matching. The propagation of electromagnetic waves in a lossy medium can be described by the vector wave equation derived from Maxwell’s equations. Solving this equation enables the prediction of the electric field distribution within biological tissues, which is crucial for evaluating antenna performance, specific absorption rate (SAR), and thermal effects in implantable devices.

Biological tissues and device materials are modeled as linear, isotropic, and locally homogeneous media with frequency-dependent complex permittivity. Metallic parts of the implant are treated as perfect electric conductors (PEC), unless finite-conductivity losses are explicitly included. At tissue and encapsulation interfaces, the tangential components of the electric and magnetic fields are continuous. At the same time, outer boundaries are terminated with a perfectly matched layer (PML) to emulate an open region and suppress reflections. These assumptions and boundary conditions ensure a well-posed electromagnetic problem and provide physically consistent electric-field distributions for subsequent SAR and thermal analyses^11,12^.

### The bioheat equation

Simultaneously with RF physics, bioheat transfer becomes a critical consideration due to the absorption of RF energy by tissues, which can lead to localized heating. Understanding the thermal effects on biological tissues is essential to ensure patient safety and compliance with regulatory standards. These effects are generally modeled using the well-established Pennes’ Bioheat Equation, which incorporates parameters such as tissue density, specific heat capacity, blood perfusion, metabolic heat generation, and thermal conductivity, as described in the literature^13,14^. In our simulation, blood temperature was assumed to be 310 K, while the ambient air temperature was set at 293.15 K. The thermal and dielectric properties used in the model are summarized in Table 2.

**Table 2:** The suggested model’s thermal and dielectric characteristics at 2.4 GHz^15,16^.

	Conductivity (σ, S/m)	Relative permittivity(*ε*_*r*_*)*	Thermal conductivity (K, W/m.K)	Density $(\rho ,$ Kg/m^3^)	Heat capacity (c_b_, J/Kg.K)
Skin	1.46	38	0.445	1109	3391
Fat	0.1	5.28	0.21	930	2770
Muscle	1.77	52.7	0.48	1100	3852
Roger 6010LM	0	ε_r_ = 10.2, tanδ = 0.0023	0.86	3100	239
Teflon	0	ε_r_ = 2.1, tanδ = 0.0002	0.24	2200	1000
Copper (annealed)	5.8 × 10^7^	1	401	8930	390

To conduct the experiment on adipose tissue, ex vivo samples of bovine intramuscular fat were sourced from local butcher shops. Ethical approval was not required, as the study did not involve live animals or any procedures performed on living subjects. The fat tissues were considered byproducts or residual materials obtained from cattle that had already been slaughtered for commercial food production. The choice of bovine adipose tissue was guided by prior studies in biomedical electromagnetics, which demonstrated that the dielectric and thermal properties of bovine fat are sufficiently close to those of human adipose tissue. These works reported that experimental measurements in bovine fat yield results that closely match simulations performed with human adipose tissue models^17^.

## The SAR

The Specific Absorption Rate (SAR) of implantable antennas refers to the electromagnetic energy absorbed by the surrounding biological tissues^17^. It is expressed as the energy absorbed per unit mass of tissue (W/kg) and is a vital indicator for evaluating the safety of implanted medical devices. The SAR depends on parameters such as the electric field (E), which represents the force exerted on an electric charge within the tissue, the electrical conductivity (σ), which measures the tissue’s ability to conduct current, and the tissue mass density (*ρ*), which reflects mass per unit volume. The mathematical formulation of SAR is well established in the literature^18,19^ and was applied in our study for performance evaluation.

To ensure the safe use of implanted devices with the proposed antenna, the SAR must comply with standard safety limits remaining below 1.6 W/kg for 1 g of tissue and 2 W/kg for 10 g of tissue^18,20^. In line with recent research, a 1 W input power is utilized for the initial SAR evaluation^20^.

### Radiation efficiency and bandwidth

Due to the interaction between the radiating elements of the antenna and human tissue, the radiation efficiency of the antenna decreases when operating inside the body. In addition, the dissipative characteristics negatively impact the antenna’s gain. Both the efficiency and gain are directly affected by the antenna’s location and orientation within the body. Furthermore, the restricted physical size of the antenna contributes to additional performance degradation. Therefore, achieving both high gain and high efficiency remains a major challenge for all implantable antennas^3^.

For in-body antennas, the majority of power is absorbed by surrounding biological tissues rather than being reflected, which causes significant absorption and partial reflection within the medium. As a result, only a fraction of the transmitted power from the source is delivered to the receiver, producing a broader operational bandwidth. This effect inevitably lowers the radiation efficiency of the antenna. To mitigate such losses and confine the bandwidth, bio-encapsulation and impedance matching techniques are commonly employed^18^.

## Results and discussion

**Fig. 4:**
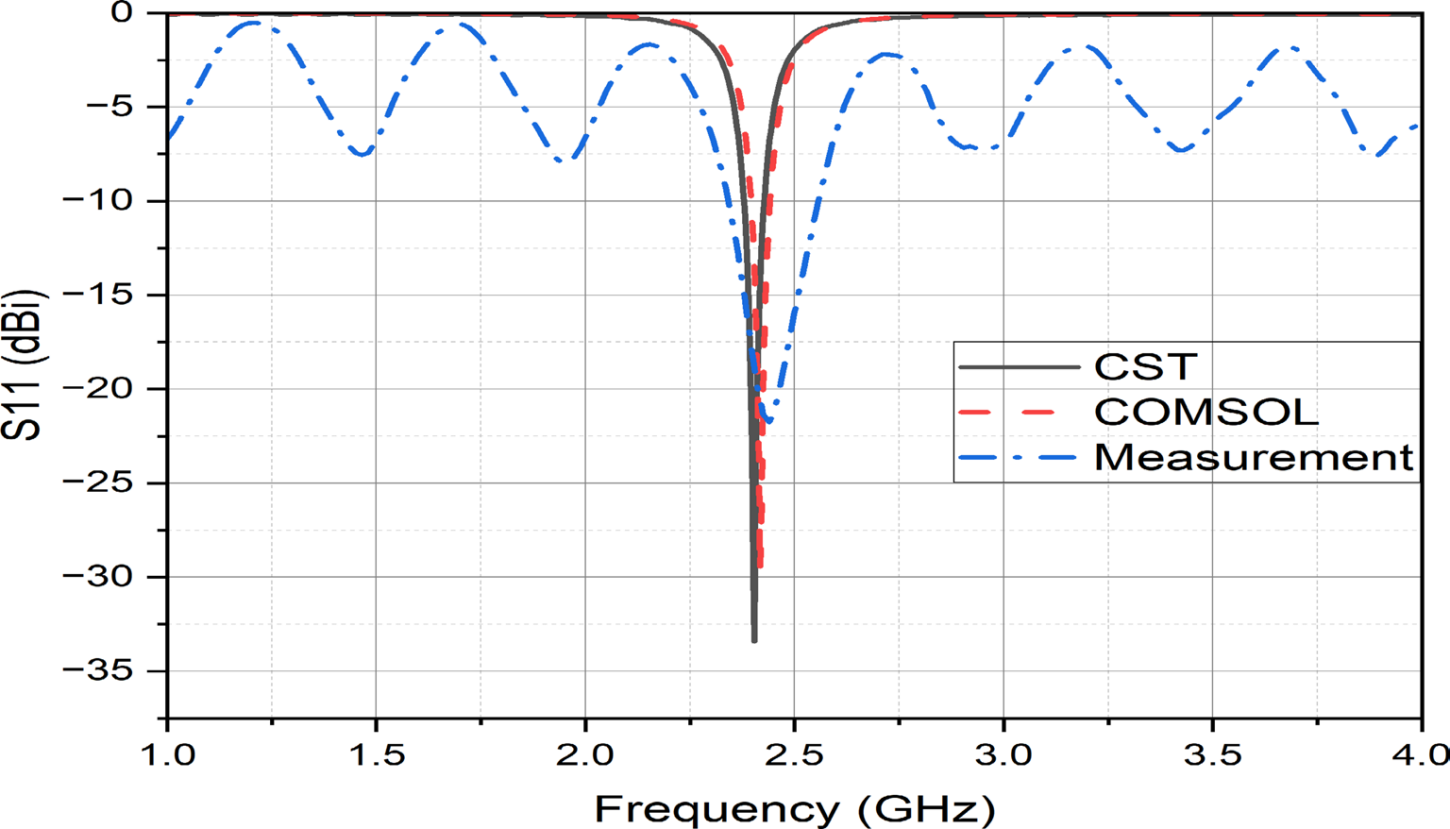
The reflection coefficient (S11) of the proposed antenna using two simulation programs with measurement.

Using the RF physics module in both CST Microwave Studio and COMSOL Multiphysics, simulations were conducted with identical parameters and dimensions to evaluate the reflection coefficient (S11), gain, 2D and 3D radiation patterns, and SAR. As illustrated in Fig. 4, the resonant frequency is identified at 2.402 GHz, which falls within the ISM band. A minor discrepancy is observed between the reflection coefficients obtained from the two platforms: approximately − 33.4 dB in CST Microwave Studio and − 29.6 dB in COMSOL Multiphysics. This difference is primarily attributed to the variation in simulation environments. The antenna was simulated inside a fat-tissue model with realistic dielectric properties at 2.402 GHz, ensuring that impedance matching accounts for strong absorption and detuning effects. Both CST and COMSOL were used, and despite slight magnitude differences, both confirmed resonance near 2.402 GHz with consistent impedance bandwidth (~ 10.4%). This indicates that the antenna maintains good matching under varied modeling environments. In COMSOL Multiphysics, a realistic surrounding medium was modeled using a Perfectly Matched Layer (PML), with the spherical region shown in Fig. 2(b) representing an air domain. In contrast, CST automatically applies the open boundary condition (PML) by default; therefore, it does not need to be manually defined as in COMSOL.

However, the bandwidth in both simulations is approximately 44.78 MHz at − 10 dB of the reflection coefficient (S11), as shown in Fig. 4.

**Fig. 5:**
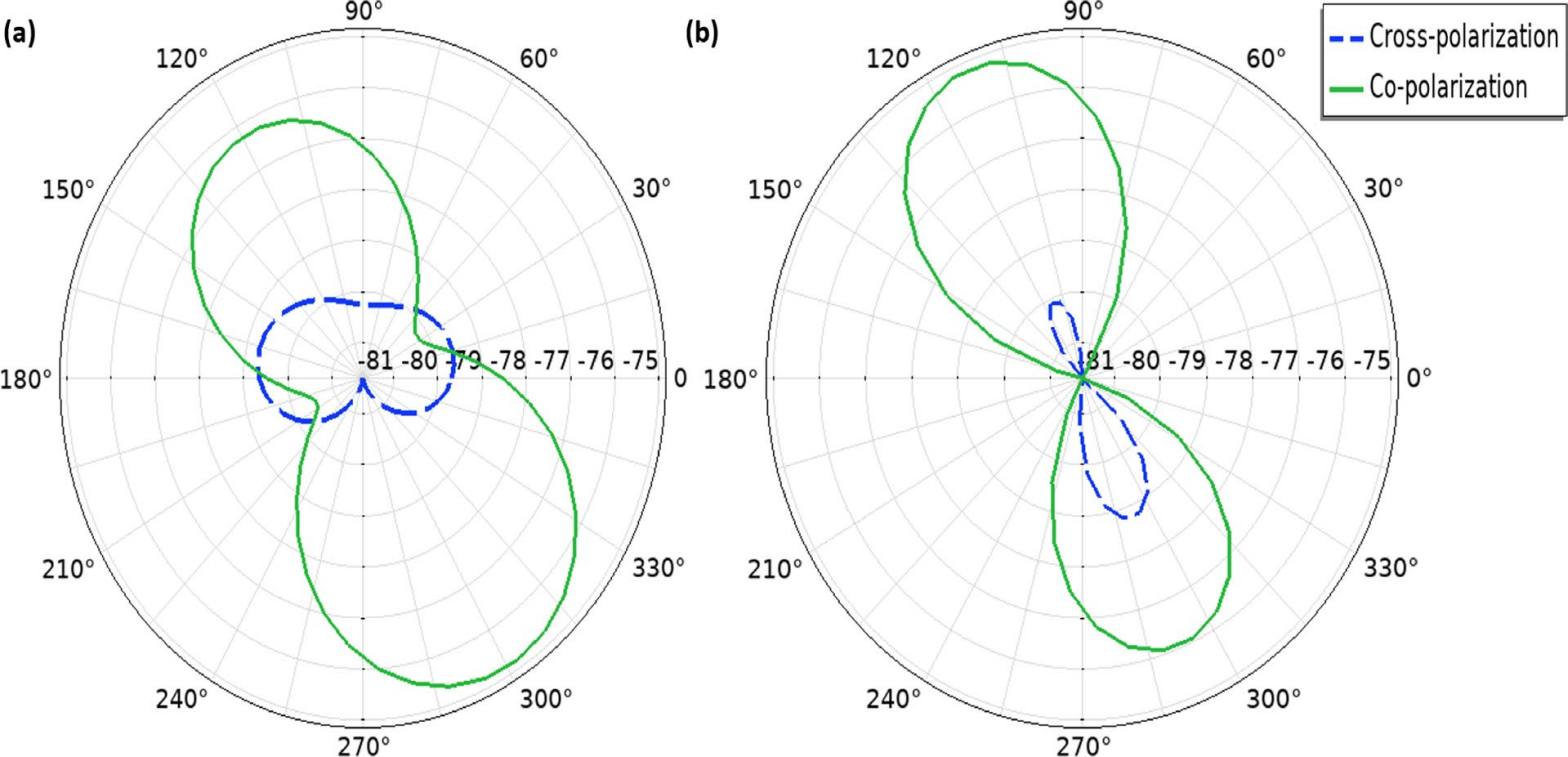
Simulated co- and cross-polarization radiation patterns of the proposed antenna using COMSOL: (**a**) E-plane; (**b**) H-plane.

The radiation patterns of the realized far-field gain (in dBi) obtained from both COMSOL and CST simulations exhibit approximately the same spatial distribution and magnitude. The maximum realized far-field gain value is − 28.3 dBi in COMSOL and − 28.2 dBi in CST. Since the antenna is designed to operate inside the human body, the surrounding biological tissues significantly affect its radiation behavior. These tissues are lossy, causing scattering and absorption of electromagnetic waves, which leads to a reduction in radiation directivity. As a result, the antenna tends to radiate in a more omnidirectional manner to ensure coverage within the body^21,22^. The E-plane refers to the plane containing both the direction of electromagnetic wave propagation and the electric field vector. It is commonly used to evaluate how the antenna radiates energy aligned with the electric field. In implantable scenarios, the E-plane is critical since it represents the primary transmission path through tissues. As shown in Fig. 5(a), the E-plane radiation pattern demonstrates strong co-polarization with minimal cross-polarization levels, confirming high polarization purity and efficient energy transfer. The antenna also exhibits notable directivity in the desired directions, which is essential for ensuring reliable intra-body communication and data transmission.

The H-plane is defined as the plane that includes the direction of wave propagation and the magnetic field vector, providing insight into radiation performance in directions orthogonal to the electric field. As illustrated in Fig. 5(b), the proposed antenna shows a well-defined co-polarized response with suppressed cross-polarization, indicating stable polarization behavior. Compared to previously reported implantable antenna structures^8,23^, the H-plane performance here maintains good directivity and reduced cross-polarization, indicating that the antenna can operate effectively even under shifts in orientation within the body. This highlights the robustness of the communication link in dynamic biological environments.

**Fig. 6:**
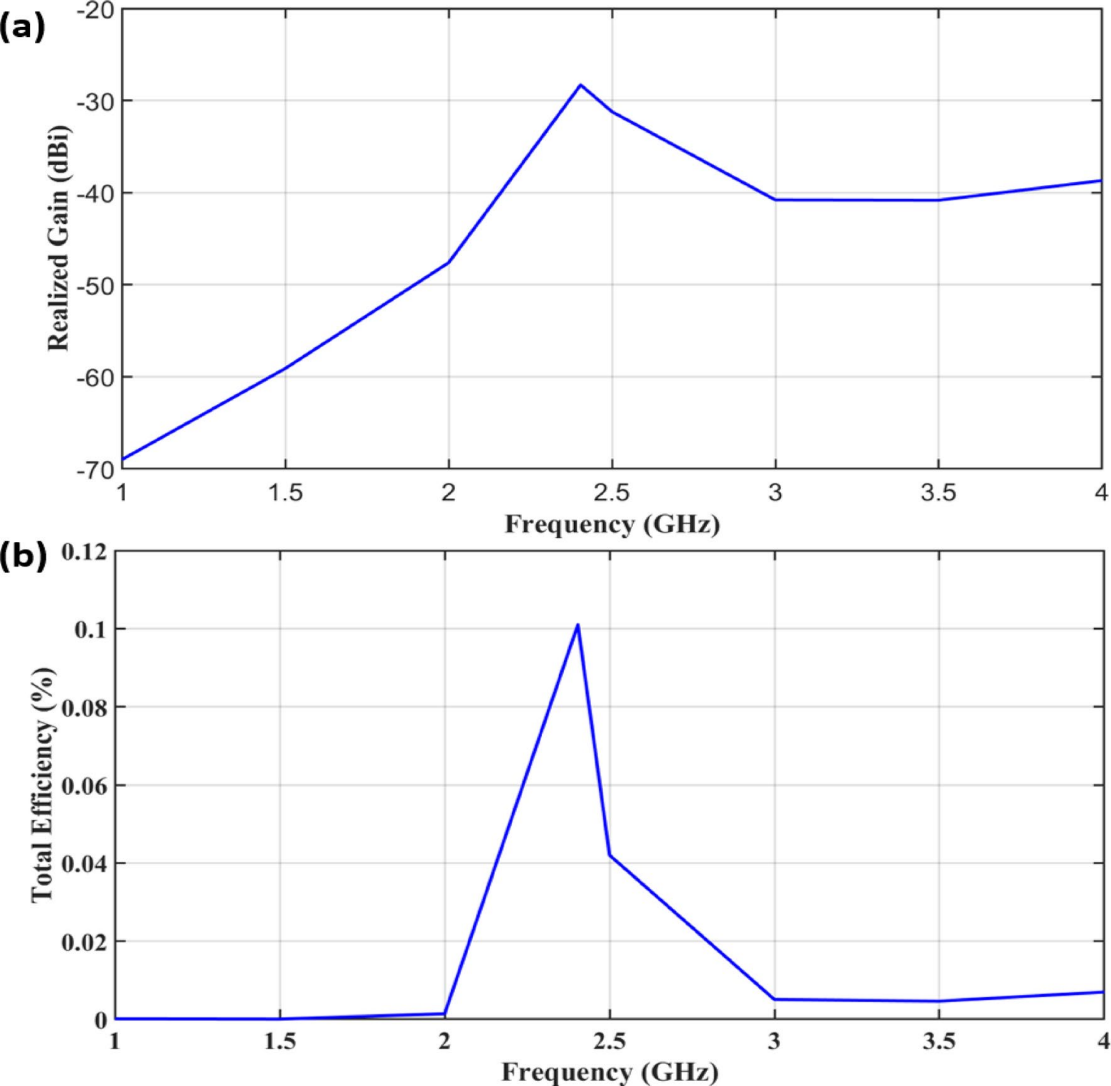
Frequency response of the proposed antenna model: (**a**) Realized gain (dBi), (**b**) Total efficiency (%).

Figure 6 illustrates the frequency response of the proposed implantable antenna in terms of realized gain and total efficiency. The results show that both parameters reach their maximum values at 2.402 GHz. Specifically, the realized gain peaks at − 28.2 dBi, while the total efficiency reaches approximately 0.102% (− 29.9 dB). In implantable antenna design, achieving high efficiency is extremely challenging due to the strong dielectric losses of biological tissues and the severe miniaturization constraints. Consequently, the total efficiency is often very low compared to conventional antennas. Negative gain values are therefore common for implantable antennas, as the surrounding tissues absorb a significant portion of the radiated energy, and the antenna operates in a complex, lossy environment rather than in free space. Reported values in the range of − 20 dB to − 40 dB are considered acceptable and consistent with prior studies on biomedical implantable devices^24^. In^25^, total efficiency was reported as 0.001% and 0.1% at 2.45 GHz using a single antenna and an arrayed antenna, respectively. Similarly, other studies have shown efficiencies as low as 0.08% to 0.1%^25^ confirming that the values achieved in this work are consistent with prior research on implantable antennas.

While the proposed antenna design was thoroughly evaluated using CST and COMSOL simulations, including reflection coefficient, gain, SAR, and thermal performance, experimental validation of the radiation pattern was not conducted due to limitations in available measurement facilities. This remains a recognized limitation of the current study. Nevertheless, the simulated radiation patterns were consistent across platforms and aligned with expected behavior for implantable antennas in lossy biological environments. Future work will focus on developing suitable measurement setups to experimentally validate the radiation characteristics and further strengthen the reliability of the proposed design.

**Fig. 7:**
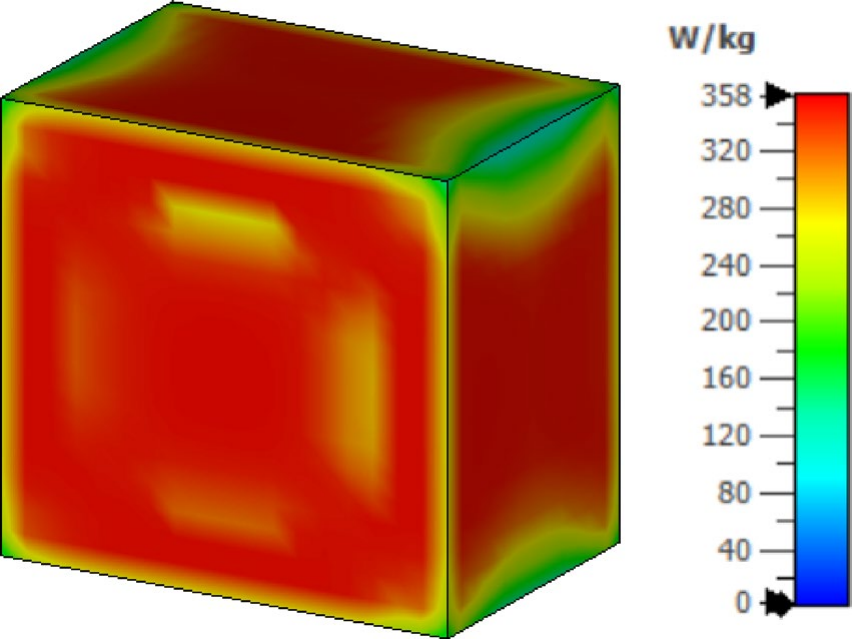
The SAR distribution in fat tissue for the proposed simulation at 1 g of tissue.

Figure 7 illustrates the effect of electromagnetic radiation on fat tissue, showing a maximum SAR value of 358 W/kg for a 1 g mass. The maximum permissible radiated power for implantable antennas is 25 µW in the MICS (402–405 MHz) and MedRadio (401–406 MHz) frequency bands, and 1 mW in the ISM band at 2.4 GHz, according to regulatory guidelines^18,20,26^. Accordingly, the SAR value calculated at 1 mW is 0.358 W/kg for 1 g of fat tissue at 2.402 GHz. When scaled to the standard input power of 1 mW typically used in implanted devices, the resulting SAR value confirms that the antenna operates well within acceptable safety limits and complies with human exposure regulations. If a design exhibits a higher SAR value, the input power is reduced to comply with practical standards. Hence, the input power is not fixed for all designs but is adjusted according to the requirements^27^. Accordingly, the maximum permitted input power of the proposed antenna is limited to 4.5 mW to meet the IEEE standards.

**Fig. 8:**
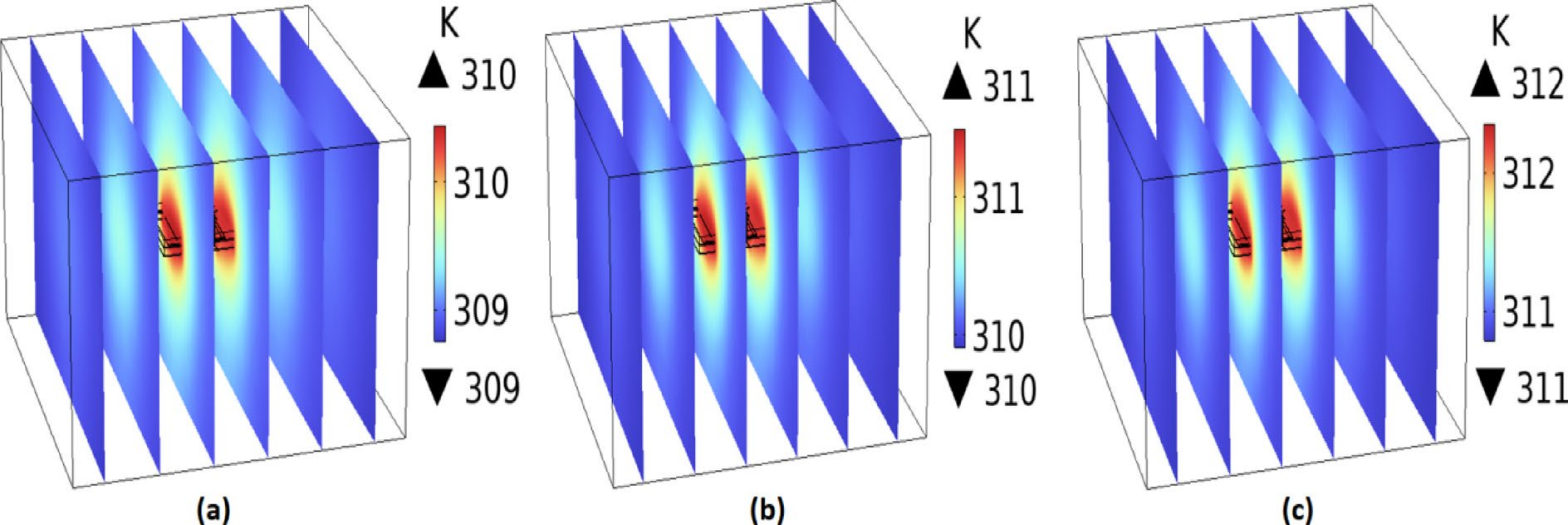
The temperature distribution on the proposed model during different times (**a**) 14 min, (**b**) 25 min, and (**c**) 35 min at the operating frequency of 2.402 GHz.

**Fig. 9:**
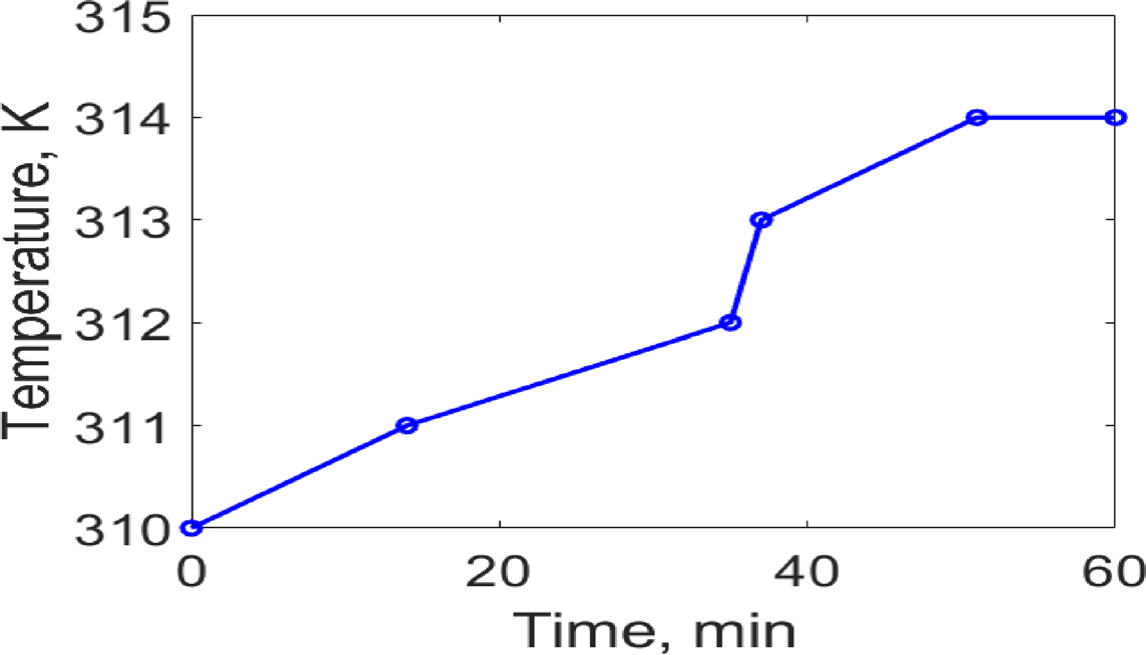
The thermal effects of the proposed implantable antenna on the surrounding biological tissue were evaluated for 60 min at the operating frequency of 2.402 GHz.

The temperature distribution was assessed using the bioheat module in COMSOL at different time intervals 14, 25, and 35 min at the operating frequency of 2.402 GHz, as presented in Fig. 8. It is evident that with prolonged exposure to electromagnetic waves in the proposed model, the ambient tissue temperature near the antenna increases by approximately 1 K between 15 and 25 min, and by an additional 2 K between 26 and 35 min. Furthermore, no noticeable temperature increase is observed during the initial 14 min of exposure. For safety considerations, the temperature increase in the tissue surrounding the implanted antenna should remain within the range of 1 to 2 K^3^. Therefore, the continuous exposure duration to the electromagnetic field should not exceed approximately 35 min to ensure safety across various biomedical applications. From our perspective, when the proposed antenna is utilized for biotelemetry applications, the continuous operation duration should not exceed 14 min to maintain the surrounding tissue temperature at 310 K. In contrast, for wireless power transfer applications, the duration may be extended up to 35 min without exceeding safe temperature limits. The temperature distribution in the surrounding biological tissue caused by the proposed implantable antenna operation was analyzed over a continuous exposure period of 60 min to assess thermal safety and compliance with biomedical standards, as shown in Fig. 9.

The continuous exposure limit of 35 min estimated in this work should be interpreted within the context of deliberately conservative assumptions, including the use of maximum regulatory input power and a 100% duty cycle (continuous-wave operation). Such conditions are unlikely to occur in realistic biomedical scenarios, where implantable devices typically operate at significantly lower power levels and rely on pulsed or intermittent transmissions. These practical operating modes substantially reduce the average thermal load on the surrounding tissue. Consequently, the reported 35-min exposure time represents a worst-case upper bound rather than a realistic operational constraint. Importantly, even under this highly conservative condition, the simulated temperature elevation in tissue remained below the widely accepted biomedical safety threshold of ΔT ≤ 2 K, thereby supporting the thermal safety of the proposed design.

Testing the antenna in real human tissue is nearly impossible; therefore, bovine intramuscular fat samples provide a practical and suitable alternative for evaluating antenna performance. In our study, the ex vivo measurements using bovine fat showed close agreement with the simulation results obtained for human adipose tissue in terms of the reflection coefficient (S11), with only minor deviations observed. These small differences are expected due to natural variability in biological tissues, but do not significantly affect the overall performance evaluation. It was observed that the reflection coefficient (S11) in the experimental measurements increased to − 22.3 dB compared to both simulation results. Additionally, the bandwidth broadened to about 0.25 GHz (10.4%), covering the range from 2.35 to 2.60 GHz, as shown in Fig. 4*.*

These discrepancies between the simulation and experimental results are mainly attributed to the quality of the SMA connector and the impact of the long coaxial cable used during the measurements.

The proposed antenna’s electromagnetic and thermal performance, including frequency, dimensions, substrate material type, bandwidth, gain, and the type of tissue used for implantation, and SAR, was compared to implantable antenna designs that had been previously mentioned in the literature, as demonstrated in Table 3.

**Table 3:** Comparison of the proposed antenna properties with the literature review.

Ref	Frequency (GHz)	Dimensions (mm^3^)	Substrate material	Gain (dBi)	SAR (W/Kg) for 1 W		Bandwidth impedance %	Implantable tissue
					1 g	10 g		
2022^5^	2.4	11.6 × 11.6 × 1.27	Rogers 3010	− 28.7	238.1	37.1	31.1	Pork phantom
2023^19^	0.86 1.43 2.6	10 × 10 × 0.125	Rogers 3010	− 26 − 14 − 16	0.409 0.534 0.529 (for 10 mW)	–	181.8 9.58 285.7	Pork muscle
2025^20^	0.915 1.4 2.45	5.8 × 6.0 × 0.254	Rogers 3003	− 31.3 − 25.8 − 21.9	320.4 332.8 464.1	32.5 34.7 49.1	152.7	Homogeneous phantom
2025^26^	1.4 2.45	3 × 4 × 0.5	Rogers 4350B	− 36 − 19.92	376.36 477.3	79.8 82.3	257 74.97	Small intestine of pork
2023^29^	2.45	8.2 × 6.94 × 0.75	Rogers 3010	− 21.6	552	73.2	16.1	Chicken
2022^30^	1.4 2.45	3.5 × 3.5 × 0.26	Rogers 3010	− 29.98 − 33.07	507 436	–	10.69 6.48	Minced pork meat
2023^31^	0.915 2.45	5 × 5 × 0.254	Rogers RT/Duroid 6010LM	− 31.2 − 23.5	–	–	21.8 40.2	Heart muscle gel phantom
2024^32^	2.45	15.5 × 4.5 × 0.085	polyimide	− 34.74	274.2	–	2.2 GHz	pork meat and phantom solution
This work	2.402	4 × 3.5 × 0.254	Rogers RT/Duroid 6010LM	− 28.2	385	–	10.4	Bovine fat

The antennas operate across a range of frequencies, mainly within the ISM and MedRadio bands (0.86 GHz to 2.6 GHz), which are suitable for biomedical telemetry. The proposed antenna in this work operates at 2.402 GHz with dimensions of 4 × 3.5 × 0.254 mm3, highlighting its suitability for implantation in small tissue areas like bovine fat. In comparison, previous works use similar compact sizes but vary widely depending on target applications.

Most antennas utilize Rogers materials such as 3003, 3010, and RT/Duroid 6010LM due to their low loss and biocompatibility. Gain values across the studies are typically negative (ranging from − 14 dBi to − 3 dBi), as expected for miniaturized implantable antennas due to high tissue absorption and limited size. The commonly acceptable gain for implantable antenna designs operating in the ISM and MICS bands is typically below − 30 dBi^28^. The proposed antenna has a gain of − 28.2 dBi, which is within the acceptable range compared to the literature.

SAR is a critical safety metric indicating how much electromagnetic energy is absorbed by body tissue. For 1 W input power, SAR values are reported at both 1 g and 10 g tissue volumes. The proposed design shows a SAR of 385 W/kg for 1 g, which is relatively high but within the trend seen in literature, especially when compared to references^26,29^, indicating strong radiation efficiency, albeit with safety considerations.

The impedance bandwidth (%), which reflects the frequency range over which the antenna maintains good impedance matching, varies widely among reported designs. The proposed antenna achieves a bandwidth of 10.4%, which is narrower than many reported works (e.g., References^20,29^), but remains acceptable depending on application requirements such as narrowband telemetry. This trade-off is intentional to achieve extreme miniaturization, ensure safe operation in lossy biological tissues, and comply with SAR and thermal constraints. Moreover, the obtained bandwidth (2.35–2.60 GHz) fully covers the target ISM channel at 2.402 GHz, while providing sufficient margin to tolerate detuning due to tissue variability and packaging. It is also important to note that several reported implantable antennas (e.g., References^19,30^) achieve even narrower bandwidths than our work, confirming that the result lies within the expected range for compact implantable devices. Therefore, the proposed antenna offers a practical balance between bandwidth, miniaturization, and safety requirements, making it particularly suitable for subcutaneous implantation applications.

The antennas are tested in various biological phantoms and actual tissues, such as a homogeneous phantom, pork tissue, chicken, and heart muscle gel. The proposed design is evaluated in bovine fat, which mimics real subcutaneous implantation conditions and demonstrates practical relevance.

Unlike previous works that mainly addressed SAR or miniaturization, the present study integrates electromagnetic and thermal analyses using a dual CST– COMSOL approach. Notably, transient thermal analysis based on Pennes’s bioheat equation was conducted, which, to the best of our knowledge, has not been reported for implantable antennas. This aspect is particularly relevant for wireless power charging applications, where continuous wave transmission may induce cumulative tissue heating, emphasizing the importance of evaluating temperature rise over time for patient safety.

Overall, the proposed antenna achieves a well-balanced performance by simultaneously addressing miniaturization, gain stability, and compatibility with realistic implant environments. Although the obtained SAR and bandwidth are slightly constrained compared to some reported alternatives, the design provides a practical trade-off that ensures electromagnetic reliability, biocompatibility, and thermal safety.

While miniaturization techniques such as shorting pins and high-permittivity substrates are well-established in the literature, the proposed antenna introduces a novel integration of these elements in a compact configuration (4 × 3.5 × 0.254 mm3), alongside a thin dielectric layer and an optimized coaxial feed. This combination enables stable impedance matching, sufficient gain (− 28.2 dBi), a 10.4% bandwidth, and compliance with SAR and thermal safety limits. Unlike previous designs that often optimize a single parameter, this work provides a balanced and comprehensive solution validated through dual-platform simulations (CST and COMSOL) and ex vivo testing.

Previous works referenced in^3^ have investigated the characteristics of implantable antennas across various biomedical applications. For leadless pacemakers, the antenna gain ranges from − 15.3 dBi to − 41.7 dBi, with a bandwidth spanning 12.8 MHz to 3380 MHz. In capsule endoscopy, the gain varies between − 16.5 dBi and − 41.41 dBi, while the bandwidth extends from 23 MHz to 13180 MHz, enabling high-data-rate transmission for imaging purposes. For intracranial pressure monitoring, the gain ranges from − 11.2 dBi to − 37.89 dBi, with a bandwidth between 18.45 MHz and 2000 MHz. These variations in antenna performance are mainly attributed to differences in antenna size, operating frequency, and substrate materials used in fabrication. Each application imposes unique constraints and requirements that significantly influence the design and electromagnetic behavior of implantable antennas.

The proposed antenna is particularly suitable for subcutaneous biomedical applications such as pacemakers, neurostimulators, biosensors, and drug delivery systems, owing to its compact size, acceptable gain, and compliance with SAR and thermal safety limits. These devices are generally implanted in shallow tissue regions, where the antenna’s performance in fat-based models and ex vivo validation closely aligns with practical requirements. Furthermore, the antenna’s bandwidth and impedance stability support low-power telemetry and control functions, which are essential for these applications^3^.

Although the proposed antenna dimensions are not the smallest reported in the literature, they were carefully optimized to achieve a trade-off between miniaturization, electromagnetic performance, thermal safety, and fabrication feasibility. The selected geometry facilitates fabrication and experimental validation, especially in integrating SMA connectors and coaxial feeds, which become increasingly challenging in ultra-miniaturized designs. Moreover, excessively small antennas often suffer from severe performance degradation, including reduced gain and narrower bandwidth, as reported in^30^. In contrast, the proposed design maintains a compact profile suitable for subcutaneous implantation while achieving a 10.4% impedance bandwidth, which is adequate for narrowband ISM biomedical applications.

Performance validation was conducted through electromagnetic and thermal simulations in both CST and COMSOL, as well as ex vivo testing using bovine fat, which served as a practical and ethically accessible surrogate for human adipose tissue. The results confirmed the antenna’s impedance stability, thermal safety (≤ 2 K rise), and capability for short-range telemetry, which meets the requirements of typical biomedical implant applications. While adipose tissue was the focus, antenna performance is expected to vary with tissue properties, with fat being the most thermally limiting case.

While long-term implantation effects were not experimentally assessed in this study, they were carefully considered during the design process. The proposed antenna is intended to be encapsulated with a biocompatible and corrosion-resistant coating such as parylene-C, medical-grade silicone, or Teflon (PTFE), which are commonly used in implantable medical devices to prevent direct contact with body fluids, reduce corrosion, and minimize biofilm formation. The selected substrate material (Rogers RT/Duroid 6010LM) and copper metallization are stable under encapsulation, but would degrade over time without a protective coating. Additionally, smooth and hydrophobic encapsulants help reduce bacterial adhesion, while the intermittent operation of the antenna—typical in telemetry systems—limits heating and further reduces the risk of tissue stress and bacterial growth. These considerations form an essential part of the proposed design’s long-term safety and reliability strategy^33^.

In this work, the current validation was limited to ex vivo testing in bovine fat, which served as a practical and ethically accessible surrogate for human adipose tissue. Future work will extend to in vivo studies in collaboration with biomedical researchers. These studies will enable assessment under dynamic physiological conditions, including blood perfusion, metabolic activity, tissue regeneration, and immune response. Key challenges include obtaining ethical and regulatory approvals, variability in tissue dielectric properties, the need for robust, biocompatible encapsulation, and the complexity of directly measuring SAR and thermal effects. These challenges can be addressed through a stepwise approach starting with small animal models, supported by biocompatibility assays, accelerated aging tests, and advanced non-invasive imaging techniques.

Although the current design was optimized for single-band ISM operation at 2.402 GHz, multi-band operation could broaden applicability to other biomedical systems such as MICS/MedRadio (402–405 MHz) and WMTS (608–1400 MHz). Designing compact, multi-band implantable antennas presents several challenges, including size constraints, dielectric loading in tissue, impedance matching trade-offs, increased SAR, and fabrication complexity. Future work may explore slot-based miniaturization, fractal geometries, or metamaterial loading to enable multi-band functionality while maintaining compactness and regulatory compliance.

Future directions also include:

- Extending analysis to multilayer and site-specific implantation scenarios.
- Studying variable duty cycles and input powers to confirm safety under broader practical conditions.
- Fabrication of the proposed antenna and experimental validation of radiation patterns and other performance metrics.
- In vivo testing to assess performance under dynamic physiological conditions, accounting for blood perfusion, metabolic activity, tissue regeneration, and immune response.
- Exploring metamaterial-enhanced efficiency or external-side beamforming to improve communication links without increasing implant size.
- Further optimization to increase gain, reduce SAR, and fine-tune bandwidth for specific biomedical applications.

The conservative 35-min continuous exposure limit ensures tissue heating remains within the 2 K threshold, providing a worst-case safety margin. The inclusion of a shorting pin and validation across multiphysics platforms contributed to robust impedance matching, and future studies will investigate variations in tissue dielectric properties to ensure stability under dynamic physiological conditions.

In summary, the proposed antenna demonstrates favorable performance, thermal safety, and miniaturization, validated through multiphysics simulations and ex vivo experiments. It is particularly suitable for subcutaneous biomedical devices requiring compact size, low power, and thermal safety. The extensive future work outlined here aims to expand functionality, validate in vivo performance, and further optimize antenna characteristics for diverse implantable medical applications.

### Effect of tissue variability on antenna performance

In the present study, simulations and measurements were conducted primarily in adipose tissue, as it represents one of the most common implantation sites for subcutaneous biomedical devices. However, dielectric and thermal properties differ considerably among tissues (e.g., muscle, skin, and fat), which in turn affect antenna performance. For instance, fat exhibits lower permittivity (ε_r_ ≈ 5.2) and conductivity (σ ≈ 0.10 S/m) compared to muscle (ε_r_ ≈ 52.7, σ ≈ 1.77 S/m) or skin (ε_r_ ≈ 38, σ ≈ 1.46 S/m). Consequently, stronger electromagnetic absorption, higher dielectric loading, and more pronounced frequency detuning with reduced efficiency and gain are expected in muscle or skin relative to fat. Regarding SAR and thermal effects, muscle tends to show higher SAR values due to its higher conductivity, but its greater perfusion facilitates heat dissipation, whereas fat—with its low conductivity and weak perfusion—presents a thermal “worst case” despite exhibiting lower absolute SAR. Impedance matching in our design is aided by the shorting pin and the high-permittivity substrate, which stabilize performance; nevertheless, minor frequency shifts (typically a few percent) may still occur in muscle or skin, consistent with prior literature^3,18^. To address these tissue-dependent variations more comprehensively, future work will extend the simulations to multilayer tissue stacks (skin–fat–muscle) and include parametric sweeps of dielectric properties (± 10–20%) to capture physiological variability across implantation sites.

**Fig. 10:**
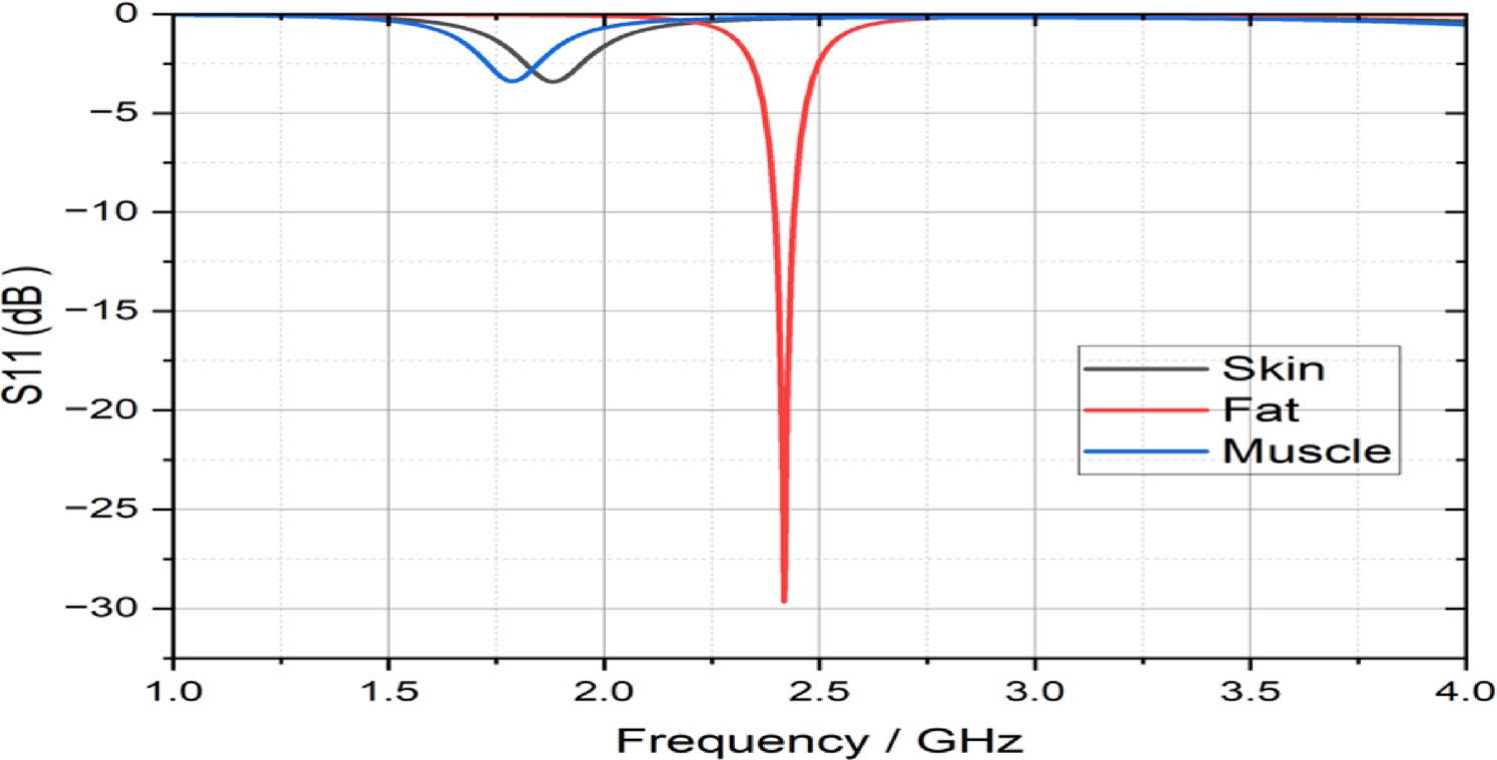
The reflection coefficient (S11) of the proposed antenna using the CST simulation program for skin, fat, and muscle.

In addition to the primary simulations in adipose tissue, further simulations were conducted by replacing the surrounding medium with skin and muscle to assess the impact of tissue variability on antenna performance. When the antenna was embedded in skin or muscle, the reflection coefficient (S11) deteriorated significantly, reaching approximately − 3.4 dB, indicating poor impedance matching as shown in Fig. 10. This outcome confirms our expectations, as the antenna was specifically designed and optimized for fat tissue properties, and its performance degrades when implanted in tissues with higher permittivity and conductivity. These results emphasize the importance of tissue-specific design and highlight the necessity for future work on multi-tissue or multilayer-optimized implantable antennas to ensure reliable performance across diverse implantation sites.

## Conclusion

This study investigated patient safety parameters of the proposed implantable antenna, focusing on SAR and thermal effects in surrounding biological tissues. A conservative 35-min continuous exposure limit was established to ensure that the temperature rise remains within the 2 K safety threshold. The inclusion of a shorting pin and validation across CST and COMSOL platforms contributed to stable impedance matching, and the antenna demonstrated acceptable performance in terms of gain, SAR, and compact size, making it suitable for subcutaneous biomedical devices such as pacemakers, neurostimulators, biosensors, and drug delivery systems.

Bovine fat was validated as a practical surrogate for experimental testing, and future studies will employ standardized human fat phantoms to improve reproducibility. While the current design is optimized for ISM band operation, future work will explore multi-band designs, multilayer and site-specific implantation scenarios, variable duty cycles and input powers, fabrication, and in vivo testing to further confirm performance and safety. Additionally, strategies such as metamaterial-enhanced efficiency or external-side beamforming may be considered to improve communication links while maintaining size and safety constraints.

## Data Availability

The data that support the findings of this study are available from the corresponding author upon reasonable request.
